# Insights into the Enhanced Tetracycline Adsorption by Two-Dimensional Cu-Based Metal–Organic Framework

**DOI:** 10.3390/molecules31050911

**Published:** 2026-03-09

**Authors:** Linteng Wang, Shi Wang, Yonglong Pang, Liyuan Guo, Jiming Huang, Ping Xue, Lingjun Kong

**Affiliations:** 1 School of Pharmacy, Xianning Medical College, Hubei University of Science and Technology, Xianning 437100, Chinapingxue@hbust.edu.cn (P.X.); 2 School of Material and Chemical Engineering, Tongren University, Tongren 554300, China; 3 Guangdong Provincial Key Laboratory of Radionuclides Pollution Control and Resources, School of Environmental Science and Engineering, Guangzhou University, Guangzhou 510006, China

**Keywords:** two-dimensional metal–organic frameworks, adsorption, tetracycline, interaction mechanism

## Abstract

Accumulation of tetracycline (TC) in aquatic environments poses a significant threat to human health and ecosystems, driving the need for efficient removal technologies. Two-dimensional metal–organic frameworks (2D MOFs) are promising adsorbents due to their tunable structures and abundant active sites. In this work, three 2D MOFs, M_3_(HHTP)_2_ (M = Cu, Ni, Co), were synthesized via a solvothermal method. Among them, Cu_3_(HHTP)_2_ exhibited superior TC adsorption with a maximum capacity of 302.84 mg/g. The adsorption process, best described by the Langmuir isotherm and pseudo-second-order kinetic models, indicates chemisorption. Mechanistic investigations reveal that the high-activity coordination sites formed by Cu^2+^ due to Jahn–Teller distortion enable strong coordination with TC. This is identified as the key factor governing the differential adsorption performance among the three MOFs. Simultaneously, the surface functional groups facilitate hydrogen bonding, and the advantageous pore structure of the material itself, together forming a synergistic adsorption. This work not only elucidates the microscopic mechanism behind the efficient adsorption of TC by Cu_3_(HHTP)_2_ but also, through comparative analysis of isostructural MOFs, confirms the decisive role of metal center electronic structure in modulating the adsorption behavior of 2D MOFs. The insights gained from this study may serve as a reference for the design of 2D high-performance adsorbents.

## 1. Introduction

Antibiotic contamination has emerged as a global environmental issue [1,2]. Tetracycline antibiotics (TC), owing to their extensive application in medical and aquaculture industries, are now ubiquitous in aquatic environments [3]. These pollutants not only promote the development of antibiotic-resistant bacteria but may also bioaccumulate through the food chain, ultimately posing threats to human health [4]. Current technologies for TC removal, including ozonation, photocatalytic degradation, microbial degradation, and membrane separation, often face limitations such as high operational cost, generation of toxic by-products, or insufficient efficiency [5]. In contrast, the adsorption method is recognized as one of the most promising strategies due to its operational simplicity, low cost, and minimal secondary pollution [6,7]. It is worth noting that the efficacy of the adsorption method depends on the development of adsorbents. Traditional adsorbents, including activated carbon, graphene oxide, and zeolite, are extensively employed for antibiotic removal owing to their high availability and low cost. Nevertheless, their further application is often hampered by intrinsic drawbacks, such as limited specific surface area, deficient functional group, and slow adsorption kinetics [8]. Therefore, the creation of novel, highly efficient, and stable adsorbent materials is crucial for achieving effective TC removal from water.

Recently, the application of two-dimensional metal–organic frameworks (2D MOFs) in pollutant adsorption has garnered significant attention due to their unique structural advantages [9]. Compared to the three-dimensional (3D) MOF, the 2D MOF possesses nanosheet architectures that expose a greater number of active sites and offer larger specific surface areas, facilitating pollutant capture [9,10]. Furthermore, the abundant π-π stacking between layers and the rich surface functional groups not only enhance the material’s stability but also enable multiple interactions with pollutant molecules, such as hydrogen bonding and π-π interactions [11]. Despite these promising prospects, systematic studies on the adsorption of TC by 2D MOFs remain relatively scarce. A comprehensive understanding of the underlying adsorption mechanisms is still lacking and requires further in-depth investigation. Notably, previous studies have shown that copper ions could coordinate with the lone pair electrons of TC to form stable TC–metal complexes accompanied by electron transfer [12]. This suggests that the nature of the metal center may play a key role in the TC adsorption. Therefore, systematically investigating the adsorption behavior of TC on 2D MOFs with different metal centers is also of great significance for revealing their structure–activity relationships.

Based on this, this work selected a series of 2D MOFs (M_3_(HHTP)_2_ (M = Cu, Ni, Co) constructed by the hexahydroxytriphenylene (HHTP) ligand as the research subject. This series of materials exhibits extended π-conjugated structures, tunable metal centers, and good stability [13], making them ideal candidates for adsorption applications. Investigating the TC adsorption behavior of M_3_(HHTP)_2_ (M = Cu, Ni, Co) can provide deeper insights into the interactions between conjugated 2D frameworks and antibiotic molecules. Moreover, their consistent topological structure helps elucidate the potential regulatory role of the metal center in the adsorption process. The findings are expected to furnish a more comprehensive foundation for the future design and screening of high-performance 2D MOF adsorbents targeting antibiotic contaminants.

## 2. Results and Discussion

### 2.1. Characterization of the Structure and Morphology of the Adsorbents

**Figure 1:**
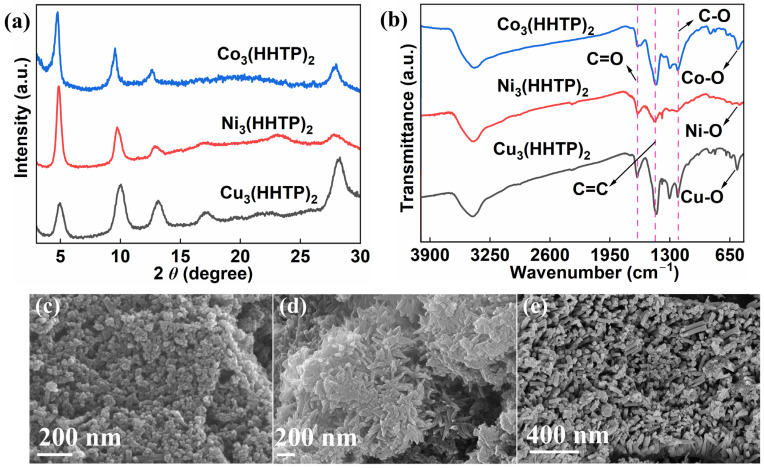
(**a**,**b**) XRD patterns (**a**) and FT-IR spectra (**b**) of Cu_3_(HHTP)_2_, Ni_3_(HHTP)_2_, and Co_3_(HHTP)_2_; (**c**,**d**) SEM images of Cu_3_(HHTP)_2_ (**c**), Ni_3_(HHTP)_2_ (**d**), and Co_3_(HHTP)_2_ (**e**).

Firstly, the morphological and structural properties of the three MOFs were systematically characterized by X-ray diffraction (XRD), Fourier-transform infrared spectrum (FT-IR), and scanning electron microscopy (SEM). XRD patterns (Figure 1a) revealed that all three MOFs exhibited a diffraction peak at 4.9°, corresponding to the (100) crystal planes [13]. For Cu_3_(HHTP)_2_ and Ni_3_(HHTP)_2_, additional peaks were observed at 9.7°, 12.9°, 17.2°, and 28°, indexed to the (200), (210), (220), and (002) crystal planes, respectively [13,14]. In the case of Co_3_(HHTP)_2_, peaks appeared at 9.6°, 12.6°, and 28°, which could be attributed to the (200), (210), and (002) crystal planes [15]. All diffraction patterns align well with previous reports, confirming the successful synthesis and high crystallinity of these MOFs. To further elucidate the structures of these adsorbents, FT-IR analysis was conducted. As illustrated in Figure 1b, the characteristic absorption bands observed at 1652 cm^−1^, 1454 cm^−1^, and 1303 cm^−1^ could be assigned to the stretching vibrations of C=O, C=C, and C-O bonds, respectively [16,17]. The appearance of the C=O bond is attributed to the in situ partial oxidation of HHTP during the reaction, leading to a redistribution of electron density on the oxygen atom, consistent with previous reports [18]. Moreover, the absorption bands near 569 cm^−1^, 545 cm^−1^, and 564 cm^−1^ corresponded to M-O (M = Cu, Ni, Co) vibrations [19,20], providing evidence of successful coordination between organic ligands and metal ions. Subsequently, the morphology of the as-synthesized MOFs was observed by SEM. From the SEM images in Figure 1c–e, it can be observed that Cu_3_(HHTP)_2_, Ni_3_(HHTP)_2_, and Co_3_(HHTP)_2_ present uniform spherical, rod-shaped particle clusters and nanorod-like morphologies, respectively. Previous studies have shown that the crystal growth direction is jointly influenced by the types of the metal precursor and the solvent environment [15,21,22]. Therefore, although all three are 2D MOFs [15,23], their microscopic morphologies exhibit significant differences. These morphologies are consistent with previous reports for the corresponding MOFs [24,25,26], further confirming the successful preparation of the materials. Moreover, the pore structure of the materials also profoundly affects their adsorption properties. N_2_ adsorption–desorption measurements revealed that all three materials exhibit type-IV isotherms with H1-type hysteresis loops [27,28] (Figure S1a–c), indicating their mesoporous structural characteristics. The Brunauer–Emmett–Teller (BET) specific surface areas of Cu_3_(HHTP)_2_, Ni_3_(HHTP)_2_, and Co_3_(HHTP)_2_ are 158, 105, and 102 m^2^/g, respectively, with Cu_3_(HHTP)_2_ having the largest specific surface area. The pore size distribution analysis (Figure S1d–f) shows that Cu_3_(HHTP)_2_ is mainly composed of micropores, while Ni_3_(HHTP)_2_ and Co_3_(HHTP)_2_ are mainly composed of mesopores. However, the average pore diameters of the three materials are 13.914, 11.582, and 12.434 nm, respectively (Table S1), which are significantly larger than their theoretical pore diameters (2 nm) [23], indicating that there are certain degrees of crystal defects in the materials [29]. Notably, the actual pore volume of Cu_3_(HHTP)_2_ is higher than the theoretical value, while that of Ni_3_(HHTP)_2_ and Co_3_(HHTP)_2_ is lower (Table S1), suggesting that defects in the latter two may lead to partial pore blockage. Additionally, the rapid uptake at very low relative pressure also indicated the coexistence of micropores within the three MOFs. This hierarchical pore structure, comprising micropores, mesopores, and macropores, facilitates the provision of abundant adsorption sites and efficient mass transfer channels for TC. Among them, Cu_3_(HHTP)_2_ has the largest specific surface area and the largest pore volume, and the two are expected to synergistically optimize its adsorption performance through enhanced mass transfer and surface binding.

### 2.2. The Adsorption Performance of the Adsorbents

**Figure 2:**
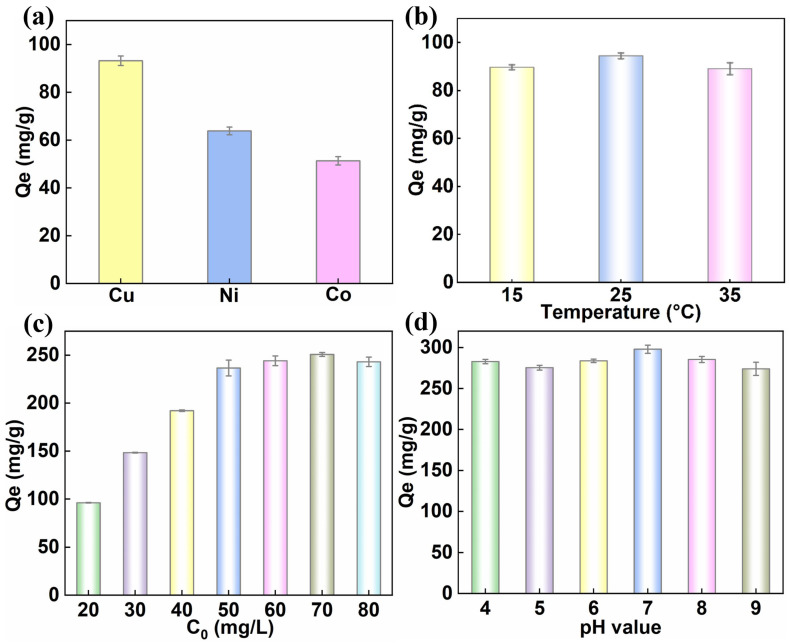
(**a**) The adsorption capacity of M_3_(HHTP)_2_ (M = Cu, Ni, Co) for TC (C_TC_ = 20 mg/L, T = 25 °C); (**b**–**d**) effects of different factors on the adsorption of TC by Cu_3_(HHTP)_2_; (**b**) temperature; (**c**) initial concentration of TC; (**d**) pH value. (Error bars represent the mean ± standard deviation (*n* = 3)).

The adsorption performance of three MOFs for TC was systematically evaluated. As expected, Cu_3_(HHTP)_2_ exhibited the most outstanding adsorption capability, achieving a removal rate of 97.32% for a 20 mg/L tetracycline solution within 60 min, which was significantly higher than those of Ni_3_(HHTP)_2_ (61.61%) and Co_3_(HHTP)_2_ (51.36%) (Figure 2a and Figure S2). In order to elucidate the adsorption behavior of Cu_3_(HHTP)_2_, the effects of various factors, including temperature, initial concentration, and pH, were investigated. These parameters were selected because temperature influences the adsorption thermodynamics, pH affects both the surface charge of the adsorbent and the speciation of TC, and the initial concentration determines the adsorption capacity [8,30,31]. Temperature experiments revealed that the maximum adsorption capacity was attained at 25 °C (Figure 2b). This optimum is likely due to insufficient molecular kinetic energy at lower temperatures, whereas at higher temperatures, the weakening of key interactions likely reduces adsorption. As for the initial TC concentration, the equilibrium adsorption capacity increased rapidly before plateauing near 70 mg/L, beyond which active site saturation and steric hindrance precluded further increase [32]. (Figure 2c). The pH value critically governs the process by modulating both the surface charge of the adsorbent and the ionic speciation of TC [33]. Within the pH range of 4–7, the negatively charged Cu_3_(HHTP)_2_ surface (Figure S3) and the zwitterionic TC molecules facilitated adsorption, which peaked under a pH value of 7 (Figure 2d). In addition, when the pH exceeded 7.7, electrostatic repulsion between the anionic TC and the negatively charged surface of the adsorbent (Figure S3) led to a decrease in capacity. It is worth noting that the adsorption capacity of TC varies little throughout the pH range of 4 to 9. Even at pH 8 (when both the adsorbent and TC carry negative charges), the adsorption capacity is still slightly higher than that at pH 4 to 6. This result indicates that electrostatic interaction is not the main driving force for adsorption. In conclusion, the optimal adsorption conditions for Cu_3_(HHTP)_2_ were identified as an initial TC concentration of 70 mg/L, a pH of 7, and a temperature of 25 °C, achieving a maximum adsorption capacity of 302.48 mg/g. Although the maximum adsorption capacity of Cu_3_(HHTP)_2_ for TC is at an intermediate-to-upper level compared to other reported MOFs (Table S2), it is worth noting that most current studies have focused on 3D MOFs. In contrast, this study provides new insights into the application of 2D MOFs in the field of antibiotic adsorption.

**Figure 3:**
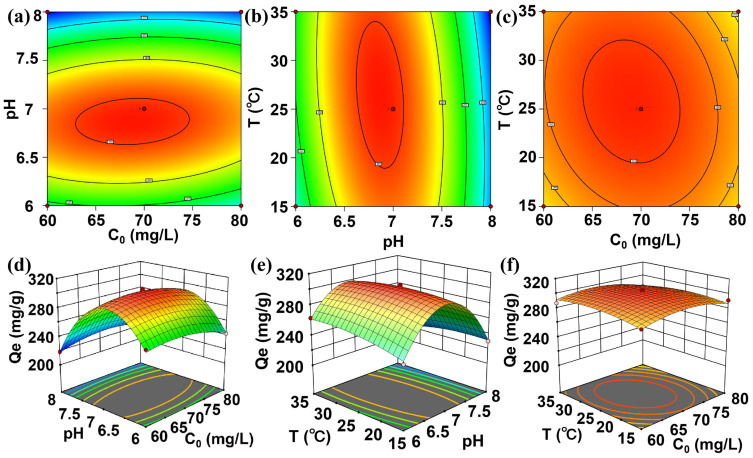
Response surface and contour plots for the effect of operating parameters on TC adsorption quantity: (**a**,**d**) factors A (initial concentration) and B (pH); (**b**,**e**) factors B (pH) and C (T); (**c**,**f**) factors A (initial concentration) and C (T).

However, adsorption is a complex process in which interactions may exist among the variables. Single-factor analysis is insufficient to fully elucidate these influence mechanisms. Therefore, to investigate the interactive effects among variables, the adsorption process was further studied by response surface methodology. A Box–Behnken design (Table S3) was employed, with the TC adsorption quantity as the response. The experimental data were fitted via multiple regression to establish a quadratic polynomial model (Equation (1)) relating the adsorption quantity to the initial concentration (A), pH (B), and temperature (C). The results indicated that the regression model was highly significant (*F* = 116.64, *p* < 0.0001), while the lack-of-fit term was not significant (*p* > 0.05) [34]. These results demonstrate a strong predictive capability of this model and minimal interference from unknown factors. Moreover, the correlation coefficient (R^2^ = 0.9954) suggested that the model aligns well with the actual data, supporting its use for analyzing and optimizing the adsorption conditions. According to Table 1, the linear term B, the interaction term BC, and the quadratic terms A^2^, B^2^, and C^2^ all showed *p*-values below 0.05, indicating their significant influence on the adsorption capacity. Among the three factors, the order of impact on TC adsorption was pH (B) > initial concentration (A) > temperature (C). The interaction effects were further examined through the response surface and corresponding contour plots (Figure 3a–f). A circular contour plot (Figure 3f) for the AC interaction indicated the absence of a significant synergistic effect between these variables. In contrast, both AB and BC produced elliptical contours (Figure 3a,b), confirming certain interactive effects. In both of these interactions, the B factor (pH) was the dominant variable (Figure 3d,e). This prominence stems from the dual role of pH in regulating the surface charge of the adsorbent and the ionization state of TC, which collectively govern the adsorption behavior. Furthermore, the model predicted the optimal adsorption conditions for TC on Cu_3_(HHTP)_2_ as follows: pH = 6.86, temperature = 26.8 °C, and initial concentration 68.55 mg/L, corresponding to a maximum theoretical adsorption capacity of 303.41 mg/g. Verification experiments conducted under these conditions yielded an actual adsorption capacity of 298.12 mg/g, which deviated from the predicted value by only 1.74%. This close agreement confirms the reliability and practical utility of the developed model.

Qe = 302.2 − 1.68A − 14.4B + 0.6924C + 4.7AB − 2.26AC − 7.66BC − 9.47A^2^ − 57.44B^2^ − 5.91C^2^ (1)

### 2.3. Cycle Experiment

The reusability and recoverability of an adsorbent are critical for its practical application. To evaluate the reusability of Cu_3_(HHTP)_2_, consecutive adsorption–desorption cycles were conducted. As shown in Figure S4a, a high retention of adsorption capacity was observed, with the material maintaining 80% of its initial uptake after five cycles. Moreover, the leaching behavior of Cu^2+^ during the adsorption process was also investigated through Inductively Coupled Plasma Optical Emission Spectrometry (ICP-OES) testing. The results (Table S4) showed that the leaching rate of Cu^2+^ was relatively low within the pH range of 4–9. Especially, at pH 7–9, the leaching rate was lower than 1%. This further confirmed the excellent stability of Cu_3_(HHTP)_2_ in aqueous solutions. Furthermore, the negligible changes in its XRD patterns (Figure S4b) and its preserved spherical morphology (Figure S4c) demonstrate the intact structure of the adsorbent after cycling. These findings collectively demonstrate that Cu_3_(HHTP)_2_ possesses not only excellent water stability but also outstanding reusability, indicating considerable potential for practical deployment.

### 2.4. Adsorption Mechanism

**Figure 4:**
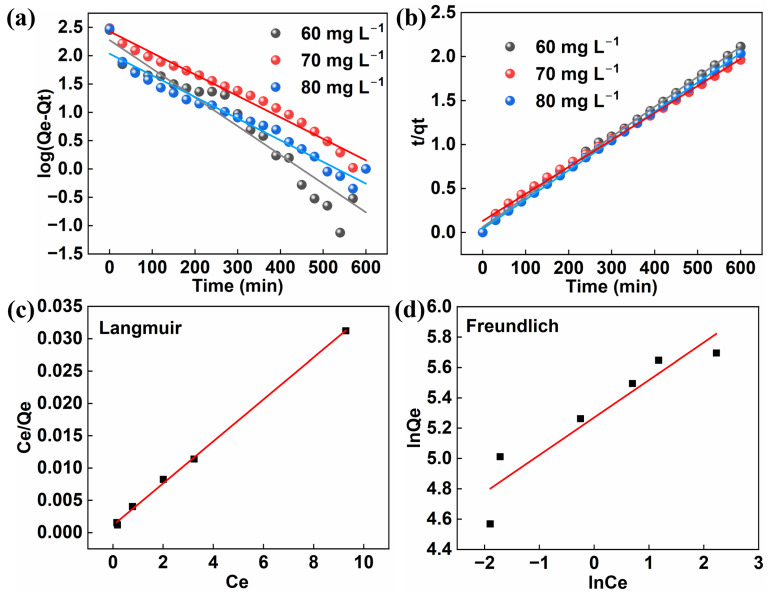
(**a**,**b**) Pseudo-first-order kinetic fitting (**a**) and pseudo-second-order kinetic fitting (**b**) of adsorption kinetics for Cu_3_(HHTP)_2_ toward TC; (**c**,**d**) Langmuir fitting (**c**) and Freundlich fitting (**d**) of the adsorption isotherms for Cu_3_(HHTP)_2_ toward TC (linear fitting).

To elucidate the adsorption mechanism of TC on Cu_3_(HHTP)_2_, the kinetic results were first analyzed. Fitted with pseudo-first-order and pseudo-second-order models, the results (Figure 4a,b and Table S5) showed that the pseudo-second-order model (60 mg/L, R^2^ = 0.9987; 70 mg/L, R^2^ = 0.9962; 80 mg/L, R^2^ = 0.9997) provided a higher correlation coefficient than the pseudo-first-order model (60 mg/L, R^2^ = 0.8977; 70 mg/L, R^2^ = 0.9768; 80 mg/L, R^2^ = 0.9552), indicating that the adsorption process might be more in line with the characteristics of chemical adsorption [35]. Further investigation of the adsorption behavior was conducted using the Langmuir and Freundlich isotherm models. As illustrated in Figure 4c,d and Figure S5 and Tables S6 and S7, the Langmuir model (linear fitting: R^2^ = 0.9984; nonlinear fitting: R^2^ = 0.9253) demonstrated a better fit compared to the Freundlich model (linear fitting: R^2^ = 0.8515; monlinear fitting: R^2^ = 0.8437). This suggests that the adsorption follows a single-layer adsorption mode [36]. This finding is highly consistent with the chemical adsorption mechanism derived from the pseudo-second-order kinetic model. Both models jointly confirm that this adsorption process is dominated by single-layer chemical adsorption and also suggest the existence of chemical bonds between Cu_3_(HHTP)_2_ and TC.

**Figure 5:**
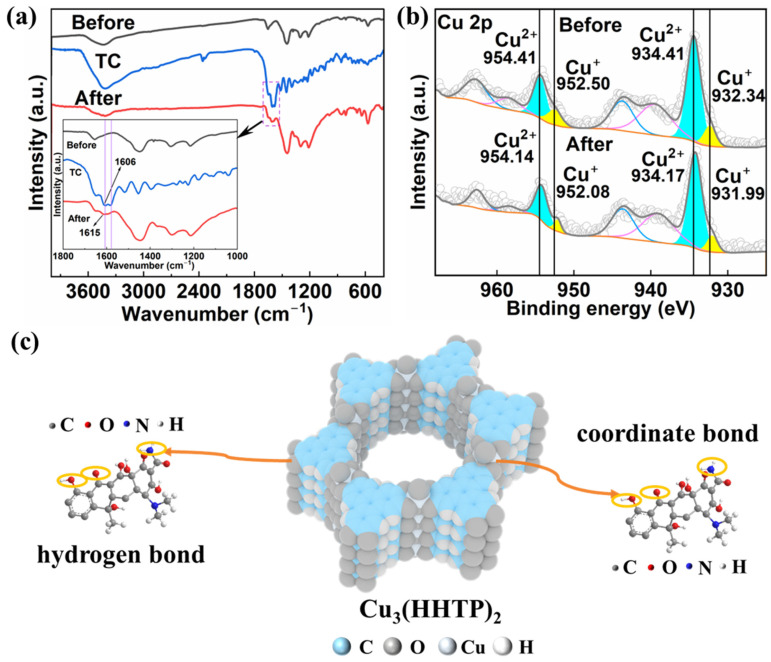
(**a**,**b**) FT-IR spectra (**a**), XPS Cu 2p spectra (**b**) of Cu_3_(HHTP)_2_ before and after adsorption of TC; (**c**) schematic diagram of the adsorption mechanism of TC on Cu_3_(HHTP)_2_.

To gain deeper insight into the adsorption mechanism, subsequent characterization was performed using FT-IR and X-ray photoelectron spectroscopy (XPS). As shown in Figure 5a, the absorption band observed at 3435 cm^−1^ in the FT-IR spectrum of Cu_3_(HHTP)_2_ can be assigned to the O-H stretching vibration of surface or residual ligand hydroxyl groups. After TC adsorption, this band exhibited a distinct red shift to 3416 cm^−1^, indicative of hydrogen bond formation between the Cu_3_(HHTP)_2_ and TC [37,38]. Furthermore, a new band emerged at 1615 cm^−1^ in the spectrum of Cu_3_(HHTP)_2_ after adsorbing TC, which was attributed to the C=O stretching vibration of TC [39]. A blue shift relative to the characteristic band of free TC (1606 cm^−1^) suggests a coordination interaction between the carbonyl oxygen of TC and the copper sites in the framework [40]. As shown in Figure 5b, the Cu 2p spectrum of Cu_3_(HHTP)_2_ before adsorption exhibits peaks at 934.41 eV and 954.41 eV, which can be attributed to Cu^2+^, while the peaks at 932.34 eV and 952.50 eV can be assigned to Cu^+^ [25]. Notably, the Cu 2p binding energy shifted to a lower value after adsorption. This shift confirms the transfer of electron density from electron-donating functional groups of TC (e.g., carbonyl, amino, or hydroxyl groups) to the copper sites and the consequent formation of coordination bonds [30,41,42], in full agreement with the FT-IR results. In summary, the adsorption of TC onto Cu_3_(HHTP)_2_ is achieved through the synergistic effects of coordination and hydrogen bonding. The combination of these interactions contributes to its high adsorption capacity (Figure 5c).

To investigate the influence of different metal centers on the adsorption performance, a comparative study was conducted on a series of M_3_(HHTP)_2_ (M = Cu, Ni, Co). The XPS results demonstrated that only the Cu 2p binding energy underwent a significant shift after TC adsorption, whereas the binding energies of Co 2p [27] and Ni 2p [43] remained virtually unchanged (Figure S6a,b). This finding proves the selectivity of the coordination interaction between TC and the metal nodes, which is a key reason for the notably higher adsorption capacity of Cu_3_(HHTP)_2_. This selectivity stems from the different metal center. The pronounced Jahn–Teller distortion of Cu^2+^ endows it with high reactivity [44], promoting strong coordination with TC. In comparison, Ni^2+^ and Co^2+^ show no significant Jahn–Teller effect and display relatively low coordination activity [45]. Consequently, the Cu center could form stable coordination bonds with TC, which substantially enhances the adsorption performance. Moreover, the Jahn–Teller distortion in Cu_3_(HHTP)_2_ also elongates the axial Cu-O bond [46]. This facilitates the complete desorption of residual solvent molecules on the surface of the synthesized material, thereby exposing more uncoordinated metal active sites. On the other hand, the previous specific surface area and pore volume analysis demonstrated that Cu_3_(HHTP)_2_ possesses a higher specific surface area and pore volume, which can provide more binding sites and mass transfer channels. This is also the key to its outstanding adsorption performance. Therefore, the superior adsorption performance of Cu_3_(HHTP)_2_ originates from the synergistic interplay between the coordination driving force provided by its metal active centers and its advantageous pore structure.

## 3. Experimental Section

### 3.1. Adsorption Experiment

The adsorption experiments of TC on the three 2D MOFs were conducted as follows: 10 mg of M_3_(HHTP)_2_ (M = Cu, Ni, Co) adsorbent was added to 50 mL of a TC solution with an initial concentration of 20 mg/L. During the adsorption process, 1 mL aliquots were collected every 10 min and filtered through a 0.22 μm filter membrane. The initial and residual concentrations of TC were determined by ultraviolet–visible (UV-vis) spectroscopy (Shanghai Jinghua, Shanghai, China, 754PC) at its maximum absorption wavelength of 358 nm. The adsorption capacity Q_t_ (mg/g), equilibrium adsorption capacity Qe (mg/g), and the removal efficiency (R) were calculated using the following formulas:

Qt = (C_0_ − C_t_) × V/W (2)

Qe = (C_0_ − C_e_) × V/W (3)

R (%) = (C_0_ − C_t_)/C_0_ × 100% (4)

(C_0_, C_t_, and C_e_ represent the initial concentration, the concentration at time t, and the equilibrium concentration of TC (mg/L), respectively; V is the volume of the TC solution (50 mL); and W is the mass of the adsorbent added (mg).)

Meanwhile, the effects of temperature, initial concentration of TC, and pH value were measured, and each adsorption experiment was conducted with a fixed stirring rate (adsorption time: 10 h). The pH of the TC solution was adjusted to the given value using 0.1 M HCl or 0.1 M NaOH.

### 3.2. Optimize Experimental Design

To determine the optimal conditions for TC adsorption onto Cu_3_(HHTP)_2_, the adsorption parameters were optimized using Box–Behnken design (BBD) based on the results of single-factor experiments. Design-Expert 13.0 software (Stat-Ease, Minneapolis, MN, USA) was employed to fit the experimental data, followed by regression and graphical analysis. The initial concentration of TC (A), initial pH value (B), and temperature (C) were selected as independent variables, while the adsorption capacity (R) was set as the response value. The BBD was applied to investigate the interactive effects of these variables on the adsorption performance.

### 3.3. Cyclic Experiment

To evaluate the reusability of Cu_3_(HHTP)_2_ for TC adsorption, adsorption–desorption cycling tests were carried out. After complete adsorption, Cu_3_(HHTP)_2_ was collected by centrifugation. Subsequently, it was immersed in ethanol and subjected to ultrasonic treatment to desorb the adsorbed TC (washing until there was no TC in the supernatant). The regenerated material was recovered by centrifugation, washed several times with ethanol and deionized water, and then reused as the adsorbent in the next cycle. The adsorption–desorption procedure was repeated five times, and the adsorption capacity in each cycle was calculated.

### 3.4. Adsorption Kinetics and Isotherms Experiments

The adsorption kinetics and isotherms experiments are the same as before. For the adsorption kinetics, the TC concentrations of 60, 70, and 80 mg/L were selected, and the pseudo-first-order and pseudo-second-order kinetic models were used to fit the adsorption kinetics data of TC to clarify the mechanism of adsorption rate control. For the isothermal line, the initial concentrations of TC were set at 20, 30, 40, 50, and 60 mg/L, with a temperature of 25 °C. The Langmuir and Freundlich isothermal adsorption models were combined to explore the adsorption behavior.

The detailed materials and reagents, synthesis of M_3_(HHTP)_2_ (M = Cu, Ni, Co), and characterization are provided in the Supporting Information. (Scheme S1 is a schematic diagram of the synthesis of M_3_(HHTP)_2_ (M = Cu, Ni, Co).)

## 4. Conclusions

This work systematically investigated the adsorption performance and mechanism of the 2D MOF-Cu_3_(HHTP)_2_ towards TC. The results demonstrated that Cu_3_(HHTP)_2_ exhibits outstanding adsorption capacity for TC, with a maximum adsorption capacity of 302.84 mg/g. The adsorption process follows the Freundlich isotherm model and the pseudo-second-order kinetic model, indicating a multilayer, heterogeneous surface-driven chemisorption. Comparative studies with Ni_3_(HHTP)_2_ and Co_3_(HHTP)_2_ further confirm the critical role of the metal center in the adsorption. XPS analysis verifies that the copper sites in Cu_3_(HHTP)_2_ engage in strong coordination with functional groups, such as the carbonyl group in TC, which is primarily attributed to the high coordination activity of Cu^2+^ induced by Jahn–Teller distortion. Moreover, the surface polar groups contribute hydrogen bonding interactions. Furthermore, a more excellent pore structure provides more binding sites and mass transfer channels. These factors collectively constitute a synergistic mechanism responsible for the efficient adsorption. This work not only elucidates the adsorption mechanism of Cu_3_(HHTP)_2_ as a high-performance adsorbent for TC but also, through comparison with isostructural MOFs, highlights the essential role of metal center electronic structure in regulating the adsorption performance of 2D MOFs. These findings provide experimental guidance for the targeted design of such materials in the future.

## Figures and Tables

**Table 1:** Analysis of variance (ANOVA) of the response surface quadratic model for TC adsorption quantity.

Source	Sum of Squares	Df	Mean Square	F-Value	*p*-Value	
**Model**	16,970.77	9	1885.64	116.64	˂0.0001	**
**A**	22.69	1	22.69	1.4	0.2748	
**B**	1659.44	1	1659.44	102.65	˂0.0001	**
**C**	3.83	1	3.83	0.2372	0.6411	
**AB**	88.48	1	88.48	5.47	0.0519	
**AC**	20.39	1	20.39	1.26	0.2984	
**BC**	234.68	1	234.68	14.52	0.0066	**
**A^2^**	377.49	1	377.49	23.35	0.0019	**
**B^2^**	13,889.66	1	13,889.66	859.17	˂0.0001	**
**C^2^**	147.22	1	147.22	9.11	0.0194	*
**Residual**	113.17	7	16.17			
**Lack of fit**	93.5	3	31.17	6.34	0.0532	Not significant
**Pure error**	19.67	4	4.92			
**Cor total**	17,083.94	16				

A: initial concentration; B: pH; C: temperature; *: significant (*p* ˂ 0.05); **: very significant (*p* ˂ 0.01).

## Data Availability

Data will be made available for reasonable request.

## Supplementary Materials

The following supporting information can be downloaded at: https://www.mdpi.com/article/10.3390/molecules31050911/s1, Figure S1: The N_2_ adsorption and desorption isotherms (a–c) and the pore size distribution (d–f) of M_3_(HHTP)_2_ (M = Cu, Ni, Co) (a and d: Cu; b and e: Ni; c and f: Co); Figure S2: Removal rates of TC by three MOFs (CTC = 20 mg/L; T = 25 °C); Figure S3. Zeta potential of Cu_3_(HHTP)_2_; Figure S4. (a) Cycle test of Cu_3_(HHTP)_2_; (b) XRD patterns of Cu_3_(HHTP)_2_ before and after adsorption of TC; (c) SEM image of Cu_3_(HHTP)_2_ after adsorption of TC; Figure S5. Langmuir fitting and Freundlich fitting of the adsorption isotherms for Cu_3_(HHTP)_2_ toward TC (Nonlinear fitting); Figure S6. XPS spectra of the (a) Co 2p of Co_3_(HHTP)_2_ and (b) Ni 2p of Ni_3_(HHTP)_2_ before and after adsorption of TC; Table S1: The BET specific surface area, pore volume, theoretical pore volume and the average pore size of M_3_(HHTP)_2_ (M = Cu, Ni, Co); Table S2. Comparison of the TC adsorption capacity of Cu_3_(HHTP)_2_ with the reported MOF-based adsorbents; Table S3. Multi-parameter experimental conditions along with observed TC adsorption capacity; Table S4. The leaching rate of Cu^2+^ in the material Cu_3_(HHTP)_2_; Table S5. Parameters of kinetic models; Table S6. Fitting parameters of adsorption isotherms (Linear Fitting); Table S7. Fitting parameters of adsorption isotherms (Nonlinear Fitting); Scheme S1. Synthesis of M_3_(HHTP)_2_ (M = Cu, Ni, Co) by HHTP with Cu(II), Ni(II) and Co(II). References [47,48,49,50,51,52,53] are cited in the Supplmentary Materials.
