# Supplementary material for: Insights into the Enhanced Tetracycline Adsorption by Two-Dimensional Cu-Based Metal–Organic Framework
Source: Molecules. 2026 Mar 9;31(5):911. doi: 10.3390/molecules31050911 (PMC12985597; doi:10.3390/molecules31050911)
Supplement: Supplementary file 1 [file molecules-31-00911-s001.zip › molecules-4116201-supplementary.pdf]

Supporting information

Insights into the enhanced tetracycline adsorption by Two-dimensional Cu-based Metal-organic framework

Linteng Wang [1, #]; Shi Wang [1, #]; Yonglong Pang [1]; Liyuan Guo [1*]; Jiming Huang [3*]; Ping Xue [1,2]; Lingjun Kong [2*]

1. School of Pharmacy, Xianning Medical College, Hubei University of Science and Technology, Xianning 437100, P. R. China

2. Guangdong Provincial Key Laboratory of Radionuclides Pollution Control and Resources, School of Envi-ronmental Science and Engineering, Guangzhou University, Guangzhou 510006, China

3. School of Material and Chemical Engineering, Tongren University, Tongren, 554300, China

#These authors contributed equally.

*Corresponding email: glyttxs@163.com; chyhjm@gztrc.edu.cn; kongljun@gzhu.edu.cn

1. Experimental section

1.1 Materials and reagents

2,3,6,7,10,11-Hexahydroxytriphenylene (HHTP, 97%) and tetracycline (TC, 98%) were purchased from Bide Pharmatech Ltd., $Cu(OAc)_2\cdot H_2O$ (99%), $Ni(OAc)_2\cdot 4H_2O$ (99%), $Co(OAc)_2\cdot 4H_2O$ (99%), *N*, *N*-dimethylformamide (DMF, A.R.), anhydrous methanol (A.R.), and anhydrous ethanol (A.R.) were purchased from Sinopharm Chemical Reagent Co., Ltd. All the materials and reagents were used directly without any further purification. Ultrapure water (18.25 MΩ·cm) was used as the experimental water source in all experiments.

1.2 Synthesis of $M_3(HHTP)_2$ (M = Cu, Ni, Co)

The synthesis of $Cu_3(HHTP)_2$ and $Ni_3(HHTP)_2$ was carried out according to the reference [1]. The synthetic methods were slightly different, mainly by changing the solvent ratio of DMF: water from 1:10 to 1:1.

The specific synthesis is as follows:

$Cu(OAc)_2\cdot H_2O$ (29.55 mg), and $Ni(OAc)_2\cdot 4H_2O$ (36.83 mg) were separately dissolved in 10 mL of a DMF/H_2O mixed solvent (1:1, v/v) to prepare three metal salt solutions. Separately, HHTP (24 mg) was dissolved in 10 mL of a DMF/H_2O mixed solvent (1:1, v/v) to obtain the ligand solution. Each metal salt solution was then mixed with the HHTP ligand solution, and the resulting mixture was stirred for 15

min. Subsequently, each mixture was transferred to reaction vessel and heated at 80°C for 12 h. After the reaction, the system was allowed to cool to room temperature, and the products were collected by centrifugation. The obtained solids were washed three times alternately with DMF and anhydrous methanol. Finally, the products were dried under vacuum at 80°C for 12 h, yielding the corresponding blue-black powders of $Cu_3(HHTP)_2$ and $Ni_3(HHTP)_2$.

The synthesis of $Co_3(HHTP)_2$ was carried out according to the reference [2]. The synthesis methods are the same.

The specific synthesis is as follows:

Dissolve HHTP (10 mg) and $Co(OAc)_2·4H_2O$ (7 mg) in 1.5 mL of H_2O. Ultrasound until the solid is completely dissolved. Then, 0.165 mL of NMP was added to the solution. Subsequently, perform ultrasound treatment to obtain a dark solution. The mixture was transferred to reaction vessel and heated at 85°C for 24 h. After the reaction, the system was allowed to cool to room temperature, and the products were collected by centrifugation. The obtained solids were washed three times alternately with deionized water and acetone. Finally, the products were dried under vacuum at 80°C for 12 h, yielding the $Co_3(HHTP)_2$.

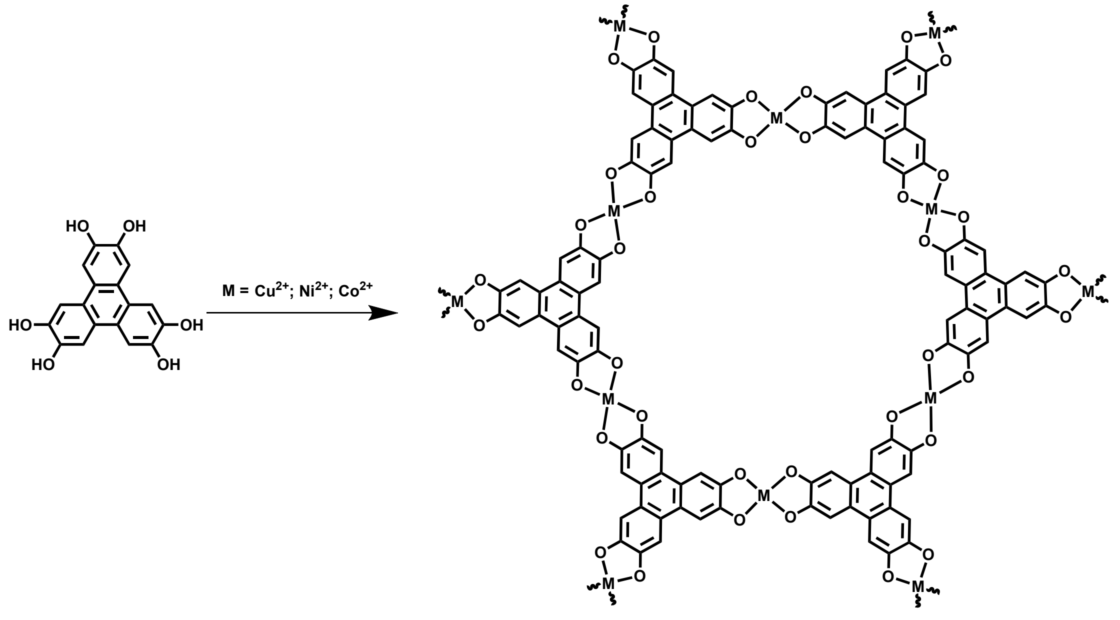

Scheme S1. Synthesis of $M_3(HHTP)_2$ (M = Cu, Ni, Co) by HHTP with Cu(II), Ni(II) and Co(II).

1.3 Characterization

The structure of these 2D MOFs was characterized by X-ray diffraction (XRD, Rigaku MinFlex 600, Japan) and Fourier-transform infrared spectroscopy (FT-IR,

Thermo Nicolet iS50, USA, using the KBr pellet method). The morphology was observed by Field-Emission Scanning Electron Microscopy (FE-SEM, Sigma 300, Germany). The specific surface area and pore structure of these 2D MOFs were evaluated from N_2 adsorption-desorption isotherms measured at 77 K using an automated specific surface area analyzer (ASAP2460-Vapor, Germany). Prior to analysis, all samples were degassed under vacuum at 80°C for 12 h. X-ray photoelectron spectroscopy (XPS, Thermo ESCALAB 250XI, American) was employed to analyze the surface elemental composition and chemical states of adsorbents. The content of Cu^{2+} in the solution after adsorption was tested by Inductively Coupled Plasma Optical Emission Spectrometry (ICP-OES, Agilent 5110, American). In addition, the surface charge of $Cu_3(HHTP)_2$ under different pH conditions was determined using a Zeta potential analyzer (Malvern Zetasizer Nano ZS90, UK). The zeta potential was determined as follows: an appropriate amount of $Cu_3(HHTP)_2$ powder was dispersed in deionized water to prepare a uniform suspension with a mass concentration of 0.1 $g{\cdot}L^{-1}$. Then, the suspension was ultrasonicated for 10 min to ensure thorough dispersion. 1 mL suspension was taken for zeta potential measurement. To ensure the reliability and repeatability of the data, each sample was measured in triplicate.

The nonlinear equation for the pseudo-first-order kinetic model:

$$Q_t = Q_e(1-e^{-k_1 t})$$

After linearization:

$$\ln{(Q_e - Q_t)} = \ln{Q_e} - k_1 t$$

The nonlinear equation for the pseudo-second-order kinetic model:

$$Q_t = \frac{k_2 Q_e^2 t}{1 + k_2 Q_e t}$$

After linearization:

$$\frac{t}{Q_t} = \frac{1}{k_2 Q_e^2} + \frac{t}{Q_e}$$

The linearized Langmuir isotherm equation:

$$\frac{C_e}{Q_e} = \frac{1}{Q_m K_L} + \frac{C_e}{Q_m}$$

The linearized Freundlich isotherm equation:

$$\ln\ Q_e = \ln\ K_F + \frac{1}{n}\ln\ C_e$$

Nonlinearized Langmuir isothermal equation:

$$q_e = \frac{q_m K_L C_e}{1 + K_L C_e}$$

Nonlinearized Freundlich isothermal equation:

$$q_e = K_F C_e^{1/n}$$

(Q_e: equilibrium adsorption capacity of TC; Q_t: adsorption capacity at time t of TC; k_1: pseudo-first-order rate constant; k_2: pseudo-second-order rate constant; C_e: equilibrium concentration of TC; K_L: Langmuir adsorption constant; K_F: Freundlich adsorption constant; n: Freundlich exponent)

Table S1. The BET specific surface area, pore volume, theoretical pore volume and the average pore size of $M_3(HHTP)_2$ (M = Cu, Ni, Co).

**Sample**	**BET specific surface area (m^2/g)**	**Pore volume (cm^3/g)**	**theoretical Pore volume (cm^3/g)**	**Average pore Size (nm)**
**$Cu_3(HHTP)_2$**	158	0.6907	0.525	13.914
**$Ni_3(HHTP)_2$**	105	0.3511	0.534	11.582
**$Co_3(HHTP)_2$**	102	0.3955	0.533	12.434

Table S2. Comparison of the TC adsorption capacity of $Cu_3(HHTP)_2$ with the reported MOF-based adsorbents.

MOF-based Sorbent	pH value	Qe ($mg\ g^{-1}$)	Interaction	Ref
MOF-5	6	233	π-π interaction	[3]
Zr-MOF-20	10	196	π-π interaction, hydrogen bonding, pore/size-selective adsorption	[4]
MIL-101-dod	-	131.17	π-π interaction, hydrogen bonding	[5]

Cu-TCPP*	9	150	Electrostatic interaction; Surface functional group interaction	[6]
Co@U-OH-1.76	8	182.31	π-π interaction, hydrogen bonding, framework coordination, electrostatic interaction	[7]
ZIF-8/NH2-MIL-53(Al)	-	578	π-π interaction, hydrogen bonding, chemisorption	[8]
Fe/Co-MOF-1/3	-	139.8	π-π interaction, hydrogen bonding, pore/size-selective adsorption, complexing action	[9]
ZnCu-MOF-74	6	775	π-interactions, metal complexation	[10]
MOF-1	10	29.78	π-π interaction, hydrogen bonding, electrostatic interaction	[11]
CYCU-3	6.5	428.14	Cation-π, π-π interaction, hydrogen bonding	[12]
MOF-525-Co	9.0	368.7	π-π interaction, hydrogen bonding, electrostatic interaction, pore-filling	[13]
$Cu_3(HHTP)_2$*	7	302.84	hydrogen bonding, coordinate bond	This work

*: Two-dimensional MOF

Table S3. Multi-parameter experimental conditions along with observed TC adsorption capacity.

Run	A-Initial concentration (mg/L)	B-pH	C-Temperature (°C)	Qe (mg/g)
**1**	60	6	25	259.331
**2**	60	8	25	242.415
**3**	80	6	25	218.762
**4**	80	8	25	220.659
**5**	70	6	15	285.928
**6**	70	8	15	291.218
**7**	70	6	35	286.926
**8**	70	8	35	283.184
**9**	60	7	15	241.267
**10**	80	7	15	230.140
**11**	60	7	35	262.874
**12**	80	7	35	221.108
**13**	70	7	25	300.200
**14**	70	7	25	299.701
**15**	70	7	25	305.040
**16**	70	7	25	302.933
**17**	70	7	25	303.094

Table S4. The leaching rate of Cu^{2+} in the material $Cu_3(HHTP)_2$.

pH value	Cu^{2+} (mg/L)	Leaching rate
4	2.042	4.38 %
5	0.797	1.71 %
6	0.499	1.07 %
7	0.322	0.69 %
8	0.307	0.66 %
9	0.208	0.45 %

Table S5. Parameters of kinetic models.

	Pseudo-first-order kinetics				Pseudo-second-order kinetics		
TC	q_e^{exp}	q_e^{cal}	K_1	R^2	q_e^{cal}	K_2	R^2
**60**	284.06	188.28	0.01167	0.8977	292.4	0.00018	0.9987
**70**	305.76	267.65	0.008728	0.9768	325.73	0.0001	0.9962

**80**	295.06	108.17	0.008797	0.9552		300.3	0.00027	0.9997

Unit of TC concentration: mg/L

Table S6. Fitting parameters of adsorption isotherms (Linear Fitting).

**T (℃)**	**Langmuir**			**Freundlich**		
	**Qm (mg/g)**	**KL**	**R2**	**1/n**	**lnKF**	**R2**
25	307.692	2.816	0.9984	0.24725	5.27048	0.8515

Table S7. Fitting parameters of adsorption isotherms (Nonlinear Fitting).

**T (℃)**	**Langmuir**			**Freundlich**		
	**Qm (mg/g)**	**K_L**	**R^2**	**1/n**	**lnK_F**	**R^2**
25	292.88mg/g	3.8	0.9253	0.1169	5.4296	0.8437

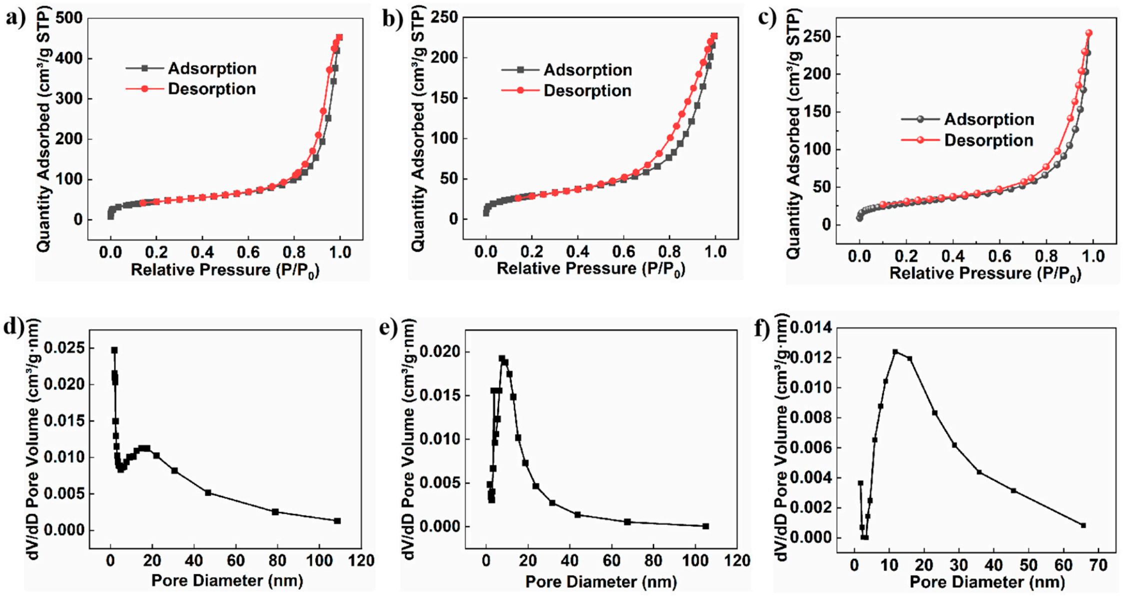

Figure S1. The N_2 adsorption and desorption isotherms (a-c) and the pore size distribution (d-f) of $M_3(HHTP)_2$ (M = Cu, Ni, Co) (a and d: Cu; b and e: Ni; c and f: Co).

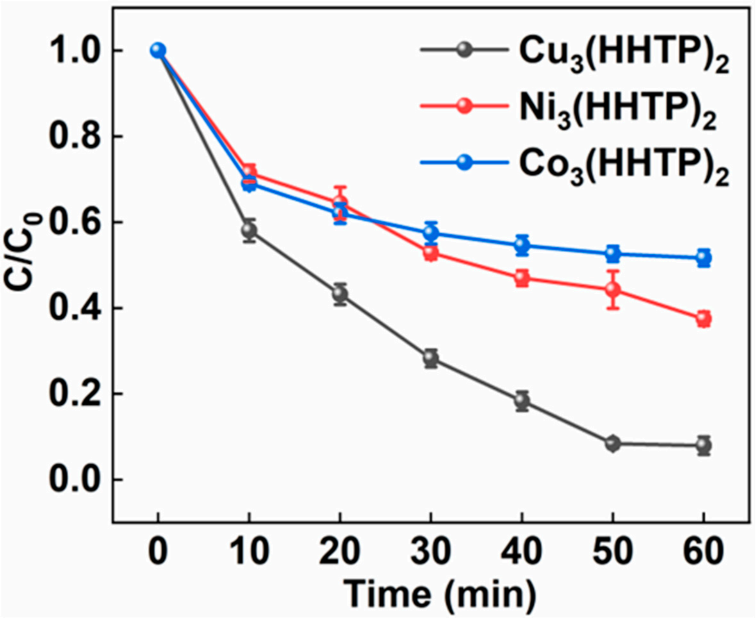

Figure S2. Removal rates of TC by three MOFs (C_{TC} = 20 mg/L; T = 25°C).

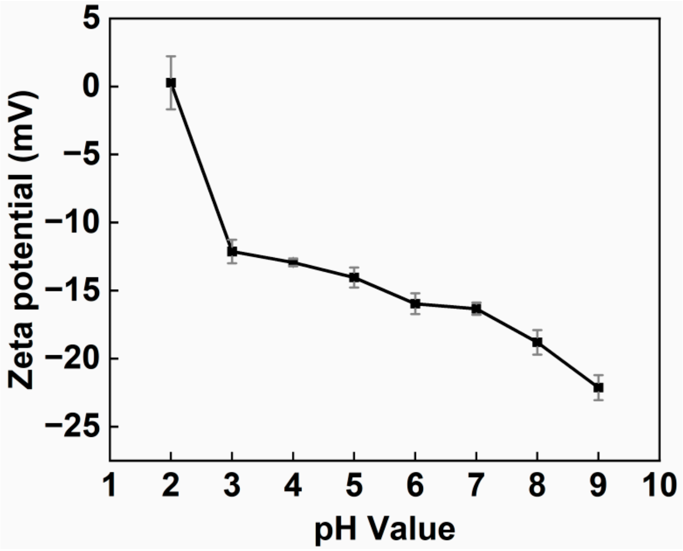

Figure S3. Zeta potential of $Cu_3(HHTP)_2$.

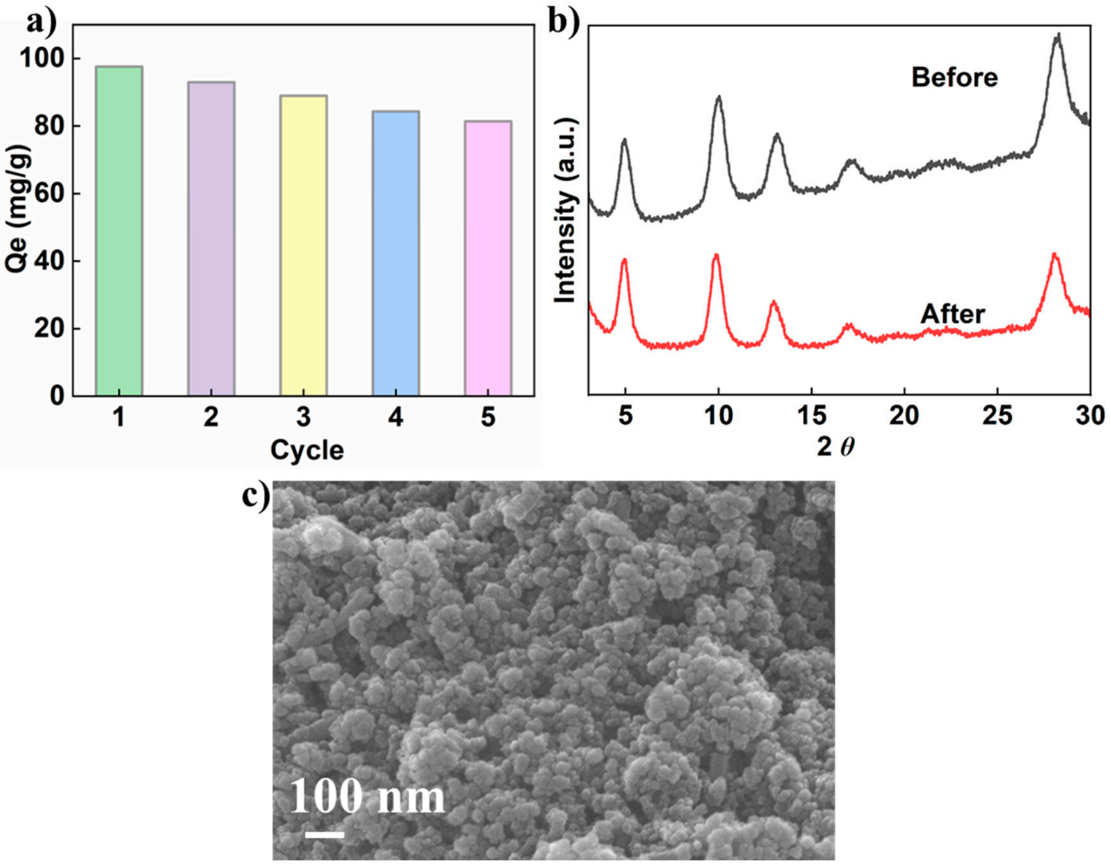

Figure S4. (a) Cycle test of $Cu_3(HHTP)_2$; (b) XRD patterns of $Cu_3(HHTP)_2$ before and after adsorption of TC; (c) SEM image of $Cu_3(HHTP)_2$ after adsorption of TC.

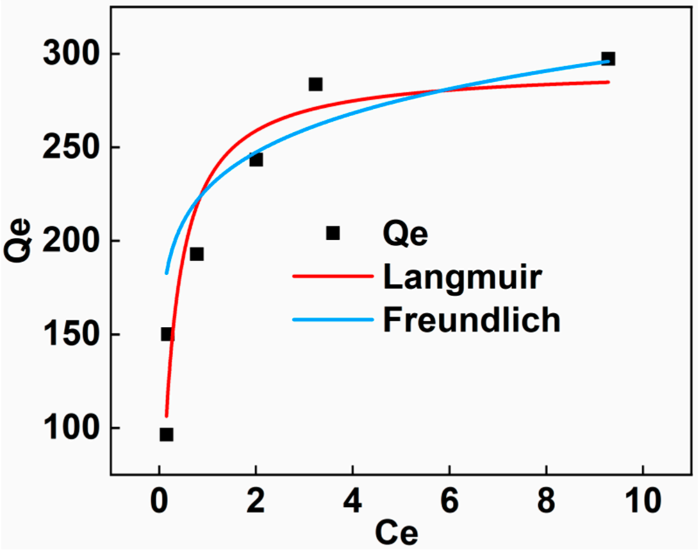

Figure S5. Langmuir fitting and Freundlich fitting of the adsorption isotherms for $Cu_3(HHTP)_2$ toward TC (Nonlinear fitting)

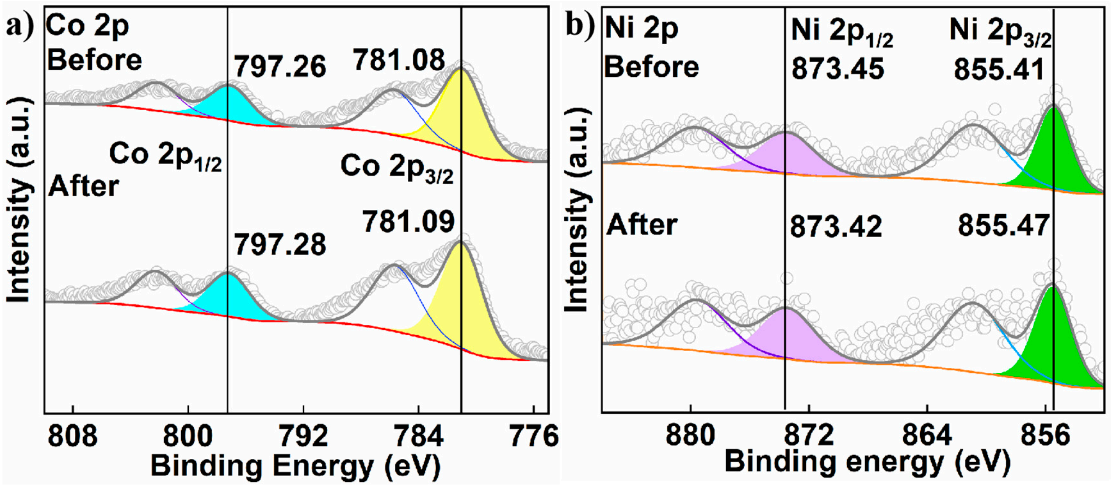

Figure S6. XPS spectra of the (a) Co 2p of $Co_3(HHTP)_2$ and (b) Ni 2p of $Ni_3(HHTP)_2$ before and after adsorption of TC.

References

1. Li, J.; Huang, Y.; Zhou, Y.; Dong, H.; Wang, H.; Shan, H.; Li, Y.; Xu, M.; Wang, X., Controllable Construction of Two-Dimensional Conductive M3(HHTP)2 Nanorods for Electrochemical Sensing of Malachite Green in Fish. *ACS Appl. Nano Mater.* **2023,** 6, (24), 22916-22926.
2. Hmadeh, M.; Lu, Z.; Liu, Z.; Gándara, F.; Furukawa, H.; Wan, S.; Augustyn, V.; Chang, R.; Liao, L.; Zhou, F.; Perre, E.; Ozolins, V.; Suenaga, K.; Duan, X.; Dunn, B.; Yamamto, Y.; Terasaki, O.; Yaghi, O. M., New Porous Crystals of Extended Metal-Catecholates. *Chem. Mater.* **2012,** 24, (18), 3511-3513.
3. Mirsoleimani-azizi, S. M.; Setoodeh, P.; Zeinali, S.; Rahimpour, M. R., Tetracycline antibiotic removal from aqueous solutions by MOF-5: Adsorption isotherm, kinetic and thermodynamic studies. *J. Environ. Chem. Eng.* **2018,** 6, (5), 6118-6130.
4. Zhong, J.; Yuan, X.; Xiong, J.; Wu, X.; Lou, W., Solvent-dependent strategy to construct mesoporous Zr-based metal-organic frameworks for high-efficient adsorption of tetracycline. *Environ. Res.* **2023,** 226, 115633.
5. Li, Y.; Peng, H.; Li, H.; Ma, Q.; Zhang, X.; Chen, Q.; Li, J.-R., Elimination of Trace Tetracycline with Alkyl Modified MIL-101 in Water. *Small* **2024,** 20, (48), 2405436.
6. Zhao, S.; Li, S.; Zhao, Z.; Su, Y.; Long, Y.; Zheng, Z.; Cui, D.; Liu, Y.; Wang, C.; Zhang, X.; Zhang, Z., Microwave-assisted hydrothermal assembly of 2D copper-porphyrin metal-organic frameworks for the removal of dyes and antibiotics from water. *Environ. Sci. Pollut. Res.* **2020,** 27, (31), 39186-39197.
7. He, S.; Zhu, J.; Tang, J.; Ji, J.; Huang, Y.; Li, Y., Monolithic Co-doped UiO-66-OH metal-organic gels for removal of tetracycline from water. *J.*

Environ. Chem. Eng. **2025,** 13, (5), 117531.
8. Li, C.; Zhang, X.; Wen, S.; Xiang, R.; Han, Y.; Tang, W.; Yue, T.; Li, Z., Interface engineering of zeolite imidazolate framework−8 on two-dimensional Al−metal−organic framework nanoplates enhancing performance for simultaneous capture and sensing tetracyclines. *J. Hazard. Mater.* **2020,** 395, 122615.
9. Wang, Z.; Wu, C.; Zhang, Z.; Chen, Y.; Deng, W.; Chen, W., Bimetallic Fe/Co-MOFs for tetracycline elimination. *Journal of Materials Science* **2021,** 56, (28), 15684-15697.
10. Flores, C. V.; Machín-Garriga, A.; Obeso, J. L.; Flores, J. G.; Ibarra, I. A.; Portillo-Vélez, N. S.; Leyva, C.; Peralta, R. A., Room-temperature synthesis of bimetallic ZnCu-MOF-74 as an adsorbent for tetracycline removal from an aqueous solution. *Dalton Transactions* **2024,** 53, (47), 18917-18922.
11. Li, K.; Li, J.-j.; Zhao, N.; Ma, Y.; Di, B., Removal of Tetracycline in Sewage and Dairy Products with High-Stable MOF. In *Molecules*, 2020; Vol. 25, p 1312.
12. Obeso, J. L.; Flores, C. V.; Boujnah, M.; Viltres, H.; Celaya, C. A.; Rosas, P. M.; Esparza-Schulz, J. M.; Ibarra, I. A.; Cordero-Sánchez, S.; Peralta, R. A.; Leyva, C., Al(III)-based MOF for tetracycline removal from water: Adsorption performance and mechanism. *J. Solid State Chem.* **2024,** 338, 124908.
13. Kong, Y.; Lu, H.; Wang, R.; Yang, Q.; Huang, B.; Zhou, Q.; Hu, W.; Zou, J.; Chen, Q., Adsorption characteristics of tetracycline hydrochloride and oxytetracycline by a MOF-525(Co) metal organic framework. *Colloids Surf. Physicochem. Eng. Aspects* **2023,** 677, 132443.
